# Genome Sequences of a Plant Beneficial Synthetic Bacterial Community Reveal Genetic Features for Successful Plant Colonization

**DOI:** 10.3389/fmicb.2019.01779

**Published:** 2019-08-13

**Authors:** Rafael Soares Correa de Souza, Jaderson Silveira Leite Armanhi, Natália de Brito Damasceno, Juan Imperial, Paulo Arruda

**Affiliations:** ^1^Centro de Biologia Molecular e Engenharia Genética, Universidade Estadual de Campinas, Campinas, Brazil; ^2^Departamento de Genética e Evolução, Instituto de Biologia, Universidade Estadual de Campinas, Campinas, Brazil; ^3^Genomics for Climate Change Research Center (GCCRC), Universidade Estadual de Campinas, Campinas, Brazil; ^4^Centro de Biotecnología y Genómica de Plantas, Universidad Politécnica de Madrid, Instituto Nacional de Investigación y Tecnología Agraria y Alimentaria (INIA), Campus Montegancedo UPM, Madrid, Spain; ^5^Instituto de Ciencias Agrarias, Consejo Superior de Investigaciones Científicas, Madrid, Spain

**Keywords:** synthetic microbial community (SynCom), plant microbiome, bacterial genome, plant–microbe association, plant-beneficial bacteria

## Abstract

Despite the availability of data on the functional and phylogenetic diversity of plant-associated microbiota, the molecular mechanisms governing the successful establishment of plant bacterial communities remain mostly elusive. To investigate bacterial traits associated with successful colonization of plants, we sequenced the genome of 26 bacteria of a synthetic microbial community (SynCom), 12 of which displayed robust and 14 displayed non-robust colonization lifestyles when inoculated in maize plants. We examined the colonization profile of individual bacteria in inoculated plants and inspected their genomes for traits correlated to the colonization lifestyle. Comparative genomic analysis between robust and non-robust bacteria revealed that commonly investigated plant growth-promoting features such as auxin production, nitrogen (N) fixation, phosphate acquisition, and ACC deaminase are not deterministic for robust colonization. Functions related to carbon (C) and N acquisition, including transporters of carbohydrates and amino acids, and kinases involved in signaling mechanisms associated with C and N uptake, were enriched in robust colonizers. While enrichment of carbohydrate transporters was linked to a wide range of metabolites, amino acid transporters were primarily related to the uptake of branched-chain amino acids. Our findings identify diversification of nutrient uptake phenotypes in bacteria as determinants for successful bacterial colonization of plants.

## Introduction

Plants and microorganisms establish mutual beneficial associations (Bonfante and Genre, 2010; Vandenkoornhuyse et al., 2015; Sánchez-Cañizares et al., 2017). By serving their hosts with a range of functions, microbes help plant growth under a variety of stressful conditions, such as drought, nutrient scarcity, and pathogen attack (Bulgarelli et al., 2013; Coleman-Derr and Tringe, 2014; Coleman-Derr et al., 2016; Gdanetz and Trail, 2017; Pineda et al., 2017). In return, plants provide its microbiota with an ample nutrient supply. Microbial communities display numerous mechanisms that allow them to identify their host, compete for resources, and establish a beneficial association (Karthikeyan et al., 2007; Lebeis et al., 2015). While the mechanisms involved in successful colonization have been widely studied in rhizobia (Desbrosses and Stougaard, 2011), it remains poorly investigated for most other plant-associated bacterial groups. Uncovering traits underlying efficient and persistent colonization has the potential to provide resources to manipulate plant microbiomes to benefit crop production under normal and stressful conditions.

Screening for selected microbial groups has guided most studies concerning the plant–microorganism interactions, with particular focus on taxonomic affiliation or known plant growth-promoting (PGP) functions such as auxin production, nitrogen (N) fixation, 1-aminocyclopropane-1-carboxylic acid (ACC) deaminase activity, and phosphorus (P) solubilization (Bulgarelli et al., 2013; Finkel et al., 2017). However, after extensive studies, these features have not been unequivocally proven to correlate with colonization efficiency or the beneficial effects observed in plants in their natural environment (Finkel et al., 2017). Also, although these approaches allow a better understanding of well-studied microbial traits, they still tend to limit the discovery of novel, undescribed functions essential for plant–microbiome interaction. In fact, investigation of plant–microbiome interactions solely based on specific functions or taxonomic affiliation does not reflect the ecological diversity and dynamics of the plant microbiota and has neglected dominant groups naturally associated with plants (de Souza et al., 2016).

Emerging strategies toward overcoming the challenges in studying complex plant microbiomes and in identifying novel functions relevant for plant microbial communities have focused on assembling synthetic or simplified microbial communities (SynCom) derived from soil or plant microbiomes (Bai et al., 2015; Lebeis et al., 2015; Timm et al., 2016; Armanhi et al., 2018; Herrera Paredes et al., 2018). By using communities instead of pure isolates, these approaches aim at partly mimicking the functional environment in which these microorganisms live (Niu et al., 2017; Vorholt et al., 2017). Importantly, they evoke the rationale of exploring microorganisms based on ecological contexts, such as their relative abundance and colonization dynamics, regardless of their taxonomic affiliation or preselected functions. However, mechanisms involved in colonization efficiency or plant growth promotion of these newly designed communities remain mostly unexplored.

We have recently described the composition of microbiota from different sugarcane organs and compartments and revealed a vast and hitherto unexplored diversity (de Souza et al., 2016). A core microbiome was identified and shown to comprise <15% of the total richness while accounting for over ∼90% of the relative abundance of bacterial and fungal operational taxonomic units (OTUs). This core microbiome was shown to be assembled and established in the different plant organs during plant development (de Souza et al., 2016). The most prevalent microbial groups comprising the core microbiome are likely the most highly adapted to live in association with the plant.

To investigate beneficial functions and colonization pattern of specific OTUs in the sugarcane core microbiome, we established a microbial culture collection of sugarcane (Armanhi et al., 2018). In order to retrieve a representative set of the microbes associated with different sugarcane organs, we decided to employ the community-based culture collection approach for large-scale isolation and identification of microbes (Armanhi et al., 2016). This procedure is based on the strategy of picking and storing colonies from a single step of plating, regardless of whether the colony contains one or multiple microbes (community-based isolates). By bypassing the several steps of picking and streaking colonies commonly employed in traditional isolation procedures, we stored multiple microbes in a single step. This procedure allowed us to establish a representative community-based culture collection that recovered a significant portion of the sugarcane core bacterial microbiome (Armanhi et al., 2016, 2018).

To test if the highly abundant groups found in the sugarcane core microbiome have beneficial effect on plant growth, we assembled a SynCom comprised of 17 community-based isolates that represent abundant bacterial groups in the sugarcane core microbiome. These isolates were selected by their high relative abundance and prevalence in sugarcane organs, regardless of their pre-existing traits or taxonomic classification. This selection was made based on the rational that highly abundant and prevalent bacteria are likely to be adapted to live in association with the plants. Inoculation of maize plants with this SynCom had an exceptional plant growth-promoting (PGP) effect (Armanhi et al., 2018). The SynCom efficiently colonized plants and accounted for 53.9, 49.1, and 9.6% of the microbial relative abundance in maize rhizosphere, endophytic root, and exophytic stem, respectively (Armanhi et al., 2018).

The cross-compatibility of the assembled sugarcane SynCom in maize, and its efficient colonization of the heterologous host, suggest that members of the SynCom encode functions that determine their fitness to live in association with plants in general. Most importantly, we noted the existence of different colonization patterns within the bacterial groups that form our SynCom. While all bacterial members of the SynCom are able to colonize plant organs, only some displayed a highly efficient colonization lifestyle and accounted for most of the relative abundance of bacteria in plant organs, thus being named as robust colonizers. In this work, we used comparative genomics to identify gene functions associated with the robust colonization lifestyle by comparing the genomes of robust and non-robust colonizers within our SynCom. Our findings point to a set of enriched functions that correlate with robust colonization and that may be essential to confer benefits to plant growth.

## Materials and Methods

### The Sugarcane Community-Based Culture Collection (CBC) and Analysis of the Colonization Pattern of the SynCom in Maize Plants

We have previously established a microbial culture collection from sugarcane containing representatives of the majority of bacterial groups detected in culture-independent surveys (Armanhi et al., 2018). This culture collection was constructed by isolating microbes from the rhizosphere, endophytic root, and endophytic stalks of mature sugarcane (*Saccharum* sp.) variety SP 80-3280. Importantly, instead of isolating axenic cultures, colonies were picked and stored regardless of being comprised by single or multiple microbes (community-based isolates). By using a multiplex amplicon sequencing method (Armanhi et al., 2016) we were able to identify the community-based isolates and retrieve information about their relative abundance in each organ of the sugarcane under natural conditions (de Souza et al., 2016). It allowed us to design a SynCom containing community-based isolates that are highly abundant in the roots and stalks of the sugarcane core microbiome (Armanhi et al., 2018). To evaluate the colonization efficiency and PGP effect of the bacteria belonging to the sugarcane core microbiome, a total of 17 community-based isolates comprising the SynCom were inoculated in 13 surface-sterilized seedlings of commercial maize (*Zea mays* L.) hybrid DKB 177 (Dekalb, Monsanto, Brazil) following the previously described protocol (Armanhi et al., 2018). Briefly, seeds were pre-germinated in sterile filter paper rolls at 28°C for 3 days in the dark. Endosperm-free seedlings were transferred to pots with vermiculite and inoculated with the SynCom. Seedlings were soaked in the inoculum for 30 min prior to transferring. Then, 1 mL of inoculum was pipetted to each seedling right after planting, 2 days and 1 week later. Plants were maintained well-watered in a greenhouse and irrigated every 3 days with 50 mL of modified Hoagland’s nutrient solution (Armanhi et al., 2018). A total of eight plants per treatment were sampled 4 weeks after planting for fresh and dry weight determination. Data were compared using Student’s *t*-test.

The microbial community profile was assessed in exophytic and endophytic compartments of roots, stems, and leaves of maize plants. Enriched microbial samples of each compartments were obtained 4 weeks after planting from five plants per treatment (inoculated and uninoculated) using methods adapted from a previously described protocol (de Souza et al., 2016). DNA extracted from enriched microbial samples using DNeasy PowerSoil Kit (Qiagen, Hilden, Germany) was used for 16S rRNA gene sequencing as previously described (de Souza et al., 2016). Raw sequence preparation and OTUs clustering from inoculated and uninoculated plants were performed with an adapted UPARSE pipeline (Edgar, 2013; de Souza et al., 2016). The colonization was identified by comparing OTU relative abundance between compartment of inoculated and uninoculated plants using FDR-corrected Kruskal–Wallis implemented in QIIME package (Caporaso et al., 2010). OTU belonging to the SynCom and presenting statistically significant differences in relative abundance between inoculated and uninoculated was considered robust colonizers.

### DNA Extraction, Library Preparation, and Genome Sequencing of Community-Based Isolates From the SynCom

The 17 community-based isolates comprising the SynCom built from a sugarcane community-based culture collection were individually grown in half-strength Luria-Bertani (LB) medium supplemented with sugarcane juice containing 8 g L^–1^ of total reducing sugars. Genomic DNA was extracted from each isolate using the Ultraclean Microbial DNA Isolation Kit (MoBio Laboratories, Carlsbad, CA, United States) following manufacturer’s protocol. An additional step of heating at 65°C for 10 min was included before vortexing. DNA was quantified by Qubit dsDNA BR Assay Kit (Thermo Fisher Scientific, Waltham, MA, United States) and its quality was validated by agarose gel electrophoresis and Nanodrop 2000c Spectrophotometer (Thermo Fisher Scientific, Waltham, MA, United States).

For library preparation, the DNA was fragmented by sonication using a Bioruptor (Diagenode, Belgium). Optimal shearing cycles were individually determined for each DNA to produce an average fragment length of 450 bp. Fragment size distribution was evaluated with an Agilent 2100 bioanalyzer (Agilent Technologies, Palo Alto, CA, United States). Sequencing libraries were individually prepared using TruSeq DNA PCR-Free Library Preparation Kit (Illumina, Inc., San Diego, CA, United States) according to the manufacturer’s protocol. Before sequencing, all libraries were quantified by qPCR with Kapa Quantification Kit (Kapa Biosystem, Boston, MA, United States) and pooled to a final equimolar concentration of 2 nM. Following denaturation, the pooled library was adjusted to 18 pM, spiked with 5% PhiX Illumina Control Library (Illumina, Inc., San Diego, CA, United States), and sequenced in an Illumina HiSeq 2500 using Rapid Mode to produce 250 bp paired-end reads.

### Read Quality Filtering and *de novo* Metagenomes Assembly

Raw paired-end reads were merged using “fastq_mergepairs” command in the USEARCH v9.2 software with parameters “-fastq_maxdiffs 10 -fastq_truncqual 4”. By using Trimmomatic v0.36 (Bolger et al., 2014), non-merged reads were filtered by a sliding window approach to cut read tails once the average Phred quality of each four bases drops below 25. Merged and filtered reads were used to assembly metagenomes with SPAdes v3.9.0 (Nurk et al., 2013). Assembly metrics were evaluated via QUAST v4.6 (Mikheenko et al., 2016).

### Individual Genome Binning and Reassembly

Individual genomes from the community-based isolates were binned and reassembly via a combined strategy of MyCC software (Lin and Liao, 2016), whole genome taxonomy classification, contig sequencing coverage, and manual curation. First, metagenomes were binned using MyCC software to cluster contigs based on marker genes and GC content. Clusters with N50 below 20,000 bp were discarded. Second, contig sequencing coverage was calculated by mapping raw, unfiltered reads onto contigs with Bowtie2 (Langmead and Salzberg, 2012). Clusters containing contigs with similar coverage values were merged. Third, contig taxonomy classification was determined using Taxator-tk software (Dröge et al., 2015). Contigs displaying divergent taxonomic classification within a cluster were discarded. Reads mapping to a particular cluster were extracted and used to reassemble individual genomes. Each extracted set of raw reads were merged, quality-filtered, and assembled by the same method described for the metagenome assembly. Scaffolds were extended using merged and filtered paired-end libraries with SSPACE (Boetzer et al., 2011). All individual genomes were evaluated for contamination and completeness to ensure high-quality assemblies using CheckM tool (Parks et al., 2015).

### Genome Annotation and Plant Growth-Promoting (PGP) Traits Prediction

The U.S. Department of Energy Joint Genome Institute (DOE-JGI) Microbial Annotation Pipeline (Mavromatis et al., 2009) was used for structural and functional genome annotation. Briefly, the pipeline executes structural annotation by searching for tRNA, rRNA, CRISPR, and coding sequences. Functional annotation was performed by assigning Cluster of Orthologous Group (COG), KEGG Orthology (KO), Pfam, and Tigrfam to every coding sequence. Annotation was maintained locally in a MySQL database for genome analyses and gene survey.

Indole-3-acetic acid (IAA) production was predicted by searching for minimal critical genes in four different biosynthetic pathways. In the “IPyA pathway”, tryptophan is converted to indole-3-pyruvate (IPyA) by a transamination, which is then decarboxylated to indole-3-acetaldehyde dehydrogenase (IAAld) by indole-3-pyruvate decarboxylase (IPDC). In the last step, IAAld is oxidized to IAA by an indole-3-acetaldehyde dehydrogenase (ALDH). Genes encoding decarboxylation (*ipdC*) and oxidation (*aldH*) have been shown to be rate-limiting for IAA biosynthesis through this pathway, and thus were used to determine the presence of this pathway in individual genomes. As for the “TAM pathway”, tryptophan is converted to tryptamine (TAM), by decarboxylation, which is then converted to IAAld by oxidation. Genes catalyzing decarboxylation (*dcc*) and oxidation (*aldH*) are critical in this pathway and were searched in bacterial genomes. In the two-step “IAM pathway”, tryptophan is converted to IAM by the enzyme tryptophan-2-monooxygenase (IaaM) and IAM is converted to IAA by IAM hydrolase (IaaH). The presence of genes encoding IaaM and IaaH was examined in the individual genomes. Finally, for the “IAN pathway”, the conversion of indole-3-acetonitrile (IAN) to IAA was investigated by looking for nitrilase genes (*nthA*).

Nitrogen fixation was predicted by searching for *nif* genes and *nif* clusters based on KO annotation. To account for the extensive variation of *nif* genes, we also searched for *nif* domains using Pfam database. Proteins containing *nif*-like domains but assigned to a function not related to N fixation were not considered for prediction.

Phosphate solubilization was predicted based on the ability of bacteria to produce gluconic acids (GAs) and phosphonate degradation and transport. GA production was determined by examining the presence of two genes based on KEGG annotation, the glucose-1-dehydrogenase (*gcd*) and gluconic acid dehydrogenase (*gad*) genes. In the case of phosphonates, genes related to phosphonates transport (*phnC*, *phnD*, and *phnE*) and degradation (*phnG*, *phnH*, *phnI*, *phnJ*, *phnK*, and *phnL*) were searched in genomes based on KEGG annotation.

ACC deaminase presence was determined by the presence of *acdS*, which encodes the 1-aminocyclopropane-1-carboxylate enzyme with deaminase activity.

### gANI and Taxonomic Affiliation of Individual Genomes

Genomic relatedness was estimated among individual genomes by calculating average nucleotide identity using orthoANI tool (Lee et al., 2016) for each genome pair. To determine genome novelty and provide an accurate taxonomic classification, the gANI value was also calculated between individual genomes from the SynCom and all the genomes belonging to the same family that were publicly available in NCBI Ref-Seq database. The taxonomic classification was performed using Taxator-tk (Dröge et al., 2015), a tool for classification of sequences based on homology.

### Analyses of Functional Enrichment/Depletion

Functional diversity analysis was performed by generating MySQL reports of gene frequency in each COG category per genome. The frequency of each COG category was calculated as the percentage of annotated genes in each category. Differentially abundant COG categories and COG protein families were identified by enrichment tests implemented as described in IMG/M’s (Chen et al., 2019) compare genome function^1^. *P*-values were corrected by false discovery rate (FDR) multiple testing.

## Results

### Members of a SynCom Display Distinct Colonization Lifestyles

**FIGURE 1:**
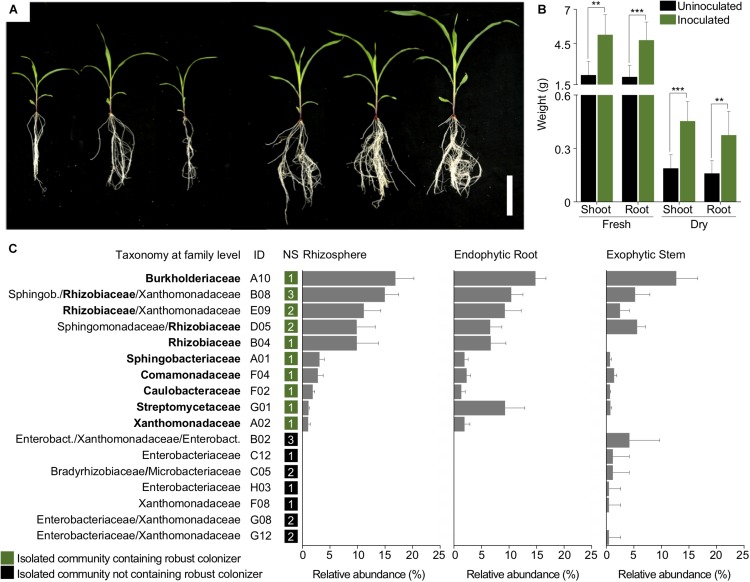
Members of a SynCom promote plant growth and show distinct colonization patterns when inoculated in maize plants. **(A)** Four-week-old maize plants’ growth in the absence (left) or presence (right) of the SynCom constituted by the 17 community-based isolates of the sugarcane community-based culture collection and **(B)** its beneficial effect in root and shoot fresh and dry weight. **(C)** Microbial composition and relative abundances of the 17 community-based isolates in inoculated maize plants. The panel shows, from left to right, the taxonomic classification; ID; number of strains (NS); and relative abundance in the rhizosphere, endophytic root, and exophytic stem for each community-isolate. A family-level classification is shown and those considered as robust colonizers are highlighted in bold. Scale bar: 10 cm. Fresh and dry weight of inoculated and uninoculated plants were compared using Student’s *t*-test (^∗∗^*p*-value ≤ 0.01; ^∗∗∗^*p*-value ≤ 0.001). Colonization was determined by difference in relative abundance of OTUs in inoculated and uninoculated plants using FDR-corrected Kruskal–Wallis test (*P* < 0.05). Sphingob., Sphingobacteriaceae; Enterobact., Enterobacteriaceae.

Maize plants were inoculated with a SynCom consisting of 17 community-based isolates from the sugarcane community-culture collection to assess their colonization profiling. The bacterial isolates used to assemble the SynCom were selected based on their high abundance in the sugarcane core microbiome (de Souza et al., 2016). Inoculation of the SynCom in maize plants reproducibly resulted in robust growth promotion, vigorous leaves and roots, and a two- to threefold increase in fresh and dry weight of shoots and roots (Figures 1A,B).

We previously demonstrated that introduction of the SynCom shifts natural microbiota in inoculated plants (Armanhi et al., 2018). Here, we explored the colonization pattern of the individual isolates from the SynCom. For this purpose, we analyzed microbial profiles through 16S rRNA gene sequencing of bacterial communities extracted from plant organs of inoculated plants. Based on 16S rRNA gene sequencing, out of the 17 community-based isolates comprising the SynCom, two were predicted to contain three bacterial OTUs, five to contain two bacterial OTUs, and the rest to contain only one OTU (Figure 1C).

The microbial profiling of inoculated plants revealed that the robust colonizers, a group of bacteria in the SynCom comprising most of the relative abundance in plant organs (Kruskal–Wallis test, *P* < 0.05), were distributed in 10 community-based isolates (Figure 1C). Among the robust colonizers, bacteria belonging to the families of Rhizobiaceae and Burkholderiaceae displayed the highest relative abundance, cumulatively accounting for 36.4% of the species in the rhizosphere, 27.6% of the endophytic compartment of the root, and 23.2% of the exophytic compartment of the stem. Interestingly, the strain belonging to the Streptomycetaceae family accounted for 7.7% of the relative abundance in the endophytic compartment of the root and represented only a small proportion in the other plant organs. Bacteria of the Comamonadaceae and Xanthomonadaceae families were found in high abundance in roots and stems, cumulatively accounting for 17.6, 14.4, and 6.7% in the rhizosphere, the endophytic compartment of root, and exophytic compartment of the stem, respectively. Although other strains in the SynCom were capable of colonizing maize plants, they were less dominant. Importantly, the contrasting difference in relative abundance between robust and non-robust colonizers was observed in all plants (*N* = 13). These results led us to question which genomic traits correlate with robust colonization lifestyle.

### Genome Sequencing of the Plant-Beneficial SynCom

To identify the genomic component that discriminates robust from non-robust colonizers and explores the genetic potential of traits associated with plant colonization by these beneficial bacteria, we sequenced individual genomic DNA from the 17 community-based isolates of the SynCom. Paired-end Illumina sequencing produced a total of 89 Gb sequence data with a read length of 250 bp. The assembly of sequencing data resulted in metagenomes with an average N50 of 839,912 bp (Supplementary Table S1). Because community-based isolates may contain one or more microorganisms, we sought to determine whether metagenomic sequencing was capable of retrieving individual genome of strains in the SynCom. The count of average hits for single-copy genes (SCGs) and search for 16S gene sequences revealed a total of 26 distinct bacterial genomes.

To reconstruct individual bacterial genomes, contigs from metagenomes of each community-based isolate were binned into clusters according to their genomic signatures, marker genes, sequencing coverage, and taxonomic affiliation. To avoid genome contamination with sequences from low represented strains or closely related species growing together in the same community isolate, we applied the binning strategy to all community isolates. Contigs that were grouped in the same cluster were assumed to belong to the same individual genome. Binning by genomic signatures and marker genes resulted in more clusters than the number of genomes initially predicted by 16S sequences and SGS analysis (Supplementary Table S2). However, within each metagenome, over 90% of the total assembly length was represented by a maximum of three clusters. The remaining fraction of the total assembly length comprised a cluster with an N50 of <2 kbp, which indicated that the larger than expected number of clusters was a result of smaller contigs that were not grouped using a clustering strategy solely based on marker genes and genomic signatures. Binning was improved by inspection of contig coverage. Because sequencing libraries were prepared with a PCR-free method, it was expected that contigs belonging to the same individual genome would display similar sequencing coverage. Thus, within each metagenome, clusters with similar sequencing coverage were merged. The binning process was validated by checking if contigs within a cluster belong to the same taxonomic classification. Contigs diverging in taxonomic affiliation were discarded. The final assembly of the community genomes resulted in a total of 26 clusters representing individual bacterial genomes (Table 1).

**TABLE 1:** Assembly statistics for the 26 individual genomes of the SynCom.

	**Total length (bp)**	**GC (%)**	**N50**	**L50**	**Largest scaffold (bp)**	**Number of contigs**	**Completeness (%)**
*Agrobacterium* sp. B08	5,139,757	58.00	661,862	3	1,763,073	12	98.21
*Agrobacterium* sp. E09	5,111,565	58.04	962,894	2	2,483,670	10	98.35
*Asticcacaulis* sp. F02	4,090,317	59.82	2,090,444	1	2,090,444	10	99.68
*Burkholderia* sp. A10	7,529,582	61.95	339,575	7	908,583	52	99.11
*Dyella* sp. G12	4,436,342	67.34	346,514	5	726,443	22	99.66
*Ensifer* sp. D05	5,733,015	62.13	24,246	74	90,054	423	85.59
*Ensifer* sp. B04	6,604,253	62.30	679,195	4	1,406,070	28	99.96
*Enterobacter* sp. B02	4,872,541	55.56	2,747,184	1	2,747,184	11	98.78
*Enterobacter* sp. F08	4,873,968	55.56	2,743,932	1	2,743,932	13	98.78
*Enterobacter* sp. G08	4,869,404	55.56	2,743,932	1	2,743,932	10	98.78
*Enterobacter* sp. G12	4,871,164	55.56	2,743,932	1	2,743,932	12	98.78
*Enterobacter* sp. H03	4,871,546	55.56	2,743,932	1	2,743,932	12	98.78
*Lysobacter* sp. A02	5,979,448	69.88	3,943,671	1	3,943,671	8	99.80
*Microbacterium* sp. C05	4,324,063	70.84	1,052,134	2	1,135,406	6	98.99
*Pantoea* sp. B02	5,004,422	57.51	533,034	3	1,656,994	18	99.98
*Pantoea* sp. C12	4,999,713	57.51	532,800	3	1,655,413	15	99.98
*Pedobacter* sp. A01	5,669,447	39.66	558,813	3	1,410,374	23	98.09
*Pedobacter* sp. B08	4,711,081	38.41	2,498,014	1	2,498,014	5	97.61
*Sphingomonas* sp. D05	3,957,378	67.26	403,020	5	611,534	27	99.66
*Stenotrophomonas* sp. B02	4,557,168	67.11	457,915	4	853,121	18	100.00
*Stenotrophomonas* sp. E09	4,341,840	67.40	465,529	4	816,873	20	100.00
*Streptomyces* sp. G01	9,718,212	70.89	648,044	6	1,225,545	28	100.00
Un. Bradyrhizobiaceae C05	6,751,956	65.62	157,875	13	432,986	81	99.53
Un. Xanthomonadaceae B08	4,846,343	69.35	3,745	329	47,962	1,594	75.23
Un. Xanthomonadaceae G08	4,766,719	64.15	1,276,390	2	1,865,598	6	100.00
*Variovorax* sp. F04	6,878,903	65.44	1,432,711	2	2,046,624	29	99.30

*Individual genomes were binned from 17 metagenomes of individual isolated communities and de novo assembled individually. Metrics for completeness was estimated using CheckM tool (Parks et al., 2015). bp, base pairs; Un., Unknown.*

Once contigs for each individual bacterial genome had been binned, all reads mapped to them were *de novo* assembled individually. *De novo* assembly resulted in individual genomes with sizes varying from 3,957,378 to 9,718,212 bp and an average N50 of 1,261,205 bp (Table 1). Low representation in the metagenomes, as determined by sequencing coverage, was the main reason for assembled genomes with an N50 below average (Table 1 and Supplementary Table S2). Final assembly quality was validated by estimating contamination and completeness with CheckM (Parks et al., 2015; Table 1). Overall, the assembled genomes showed over 98% completeness and <2% contamination (Table 1).

### Taxonomic Affiliation and Genome Relatedness

Taxonomic assignment based on whole-genome and 16S sequences showed that the assembled individual genomes span 16 genera distributed among 10 different families (Supplementary Table S3). Although most of the assembled genomes belong to Proteobacteria phylum, there are two representatives of the Actinobacteria and two of the Bacteroidetes phyla (Supplementary Table S3). The Enterobacteriaceae is the most represented family, with seven members, followed by Xanthomonadaceae and Rhizobiaceae, with six and four members, respectively (Supplementary Table S3).

**FIGURE 2:**
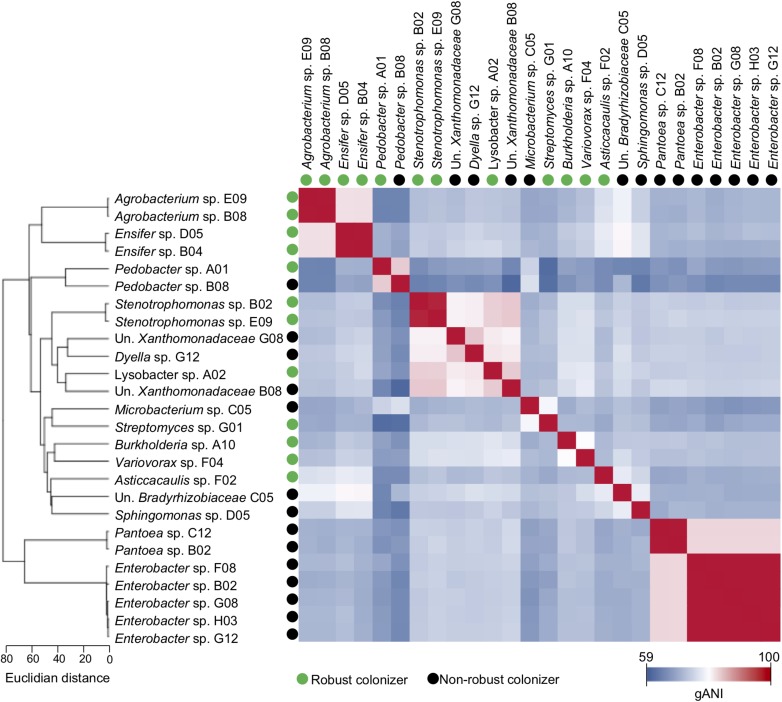
Robust colonization lifestyle is found in genetically distant bacteria. The SynCom is comprised of 17 community-based isolates harboring 26 strains that span 16 different genera. Calculation of whole-genome identity between pairs of genomes revealed no correlation between robust colonization lifestyle and genome relatedness. Distance and relatedness of bacterial genomes were calculated based on whole-genome average nucleotide identity (gANI) between each pair of genomes.

To estimate the genetic relatedness of the individual assembled genomes, we calculated pairwise whole-genome average nucleotide identity (gANI) of individual genomes (Figure 2). The gANI plot revealed that, despite the fact that the five genomes belonging to the genus *Enterobacter* came from different community-based isolates, they exhibited nucleotide identity above 99%, which indicates that they belong to the same species. A particular case was found for the two *Ensifer* genomes *Ensifer* sp. B04 and *Ensifer* sp. D05, which differ in size by 0.87 Mbp (Table 1) but displayed a 99% identity for the rest of their genomes. It is likely that the genome size divergence between these two isolates was the result of the lower representativeness of *Ensifer* sp. D05 in its metagenome, which might have negatively impacted the genome binning and reassembly process (Supplementary Table S2). Notably, despite the divergence in genome size, both *Ensifer* sp. genomes were estimated to have a 98.8% completeness. Conversely, the *Agrobacterium* sp. E09 and *Agrobacterium* sp. B08 genomes showed a gANI value over 99%, which is consistent with the genome size and completeness of both bacteria (Figure 2 and Supplementary Table S2).

Finally, the Euclidean distance based on nucleotide identity of the assembled genomes demonstrated high genetic distance among the bacterial groups of the SynCom (Figure 2). This result suggests a high functional and phenotypic versatility within the community. Importantly, we did not observe any significant correlation between robust colonization and genome relatedness (Figure 2). In fact, the broad phylogenetic affiliation and genetic divergence of robust colonizers indicate that success in colonization is an adaptive trait that might be related to functional convergent evolution.

### Individual Genome Annotation

The functional diversity among the bacterial groups of the SynCom was assessed by functional and structural annotation of individual genomes using the JGI microbial genome annotation pipeline (Huntemann et al., 2015; Table 2). Although the number of genes differed significantly between individuals of the different bacterial families, ranging from 3,739 to 8,864 genes, the coding density was similar among genomes, averaging 89% (Table 2). On average, 82% of the proteins encoded by the bacterial genomes were confidentially assigned to a functional group, and 73% were mapped to COG or KEGG orthologous groups.

**TABLE 2:** Structural and functional features of the 26 individual genomes of the SynCom.

	**Protein coding genes**		**tRNA**	**rRNA**	**Other RNAs**	**CRISPR elements**	**Protein with**				**Orthologs**	**Genes coding enzymes**	**Genes w/o function prediction (%)**
									
	**Number**	**Density (%)**					**COG**	**KO**	**pfam**	**tigrfam**			
*Agrobacterium* sp. BO8	4,856	88.4	46	1	14	1	3,683	2,573	4,102	1,367	3,460	1,222	16.70
*Agrobacterium* sp. E09	4,831	88.4	45	1	14	1	3,672	2,568	4,089	1,365	3,450	1,222	16.58
*Asticcacaulis* sp. F02	3,739	90.0	45	4	10	0	2,486	1,829	3,024	1,073	2,257	1,001	20.46
*Burkholderia* sp. A10	6,859	87.7	51	11	16	0	4,970	3,359	5,752	1,911	3,111	1,705	17.09
*Dyella* sp. G12	3,862	89.8	46	5	7	3	2,714	1,983	3,237	1,262	2,683	1,024	16.99
*Ensifer* sp. D05	5,760	86.6	54	8	21	0	3,961	2,611	4,706	1,352	3,781	1,239	19.60
*Ensifer* sp. B04	6,209	87.1	53	3	19	0	4,721	3,153	5,286	1,584	4,042	1,516	16.57
*Enterobacter* sp. B02	4,704	89.7	69	13	70	0	3,647	3,026	4,181	1,817	3,546	1,440	10.54
*Enterobacter* sp. F08	4,704	89.7	71	15	70	0	3,648	3,027	4,182	1,817	3,546	1,440	10.46
*Enterobacter* sp. G08	4,701	89.7	71	12	70	0	3,648	3,027	4,180	1,817	3,546	1,440	10.51
*Enterobacter* sp. G12	4,703	89.7	71	13	70	0	3,647	3,026	4,182	1,817	3,546	1,439	10.48
*Enterobacter* sp. H03	4,702	89.7	71	14	70	0	3,648	3,027	4,182	1,817	3,545	1,440	10.44
*Lysobacter* sp. A02	4,941	85.8	60	4	6	2	3,013	2,081	3,734	1,258	2,915	1,068	25.62
*Microbacterium* sp. C05	3,914	93.0	48	6	7	0	2,660	1,755	3,234	903	2,089	941	17.30
*Pantoea* sp. B02	4,759	88.1	69	10	57	0	3,612	2,934	4,149	1,700	3,452	1,362	12.23
*Pantoea* sp. C12	4,756	88.1	67	10	57	0	3,611	2,934	4,150	1,700	3,453	1,362	12.20
*Pedobacter* sp. A01	4,906	88.3	52	6	105	3	2,472	1,607	3,594	1,142	2,697	911	24.85
*Pedobacter* sp. B08	4,129	88.1	46	4	36	1	2,353	1,513	3,192	1,031	2,602	848	22.67
*Sphingomonas* sp. D05	3,783	90.1	47	3	8	0	2,464	1,804	2,988	1,104	2,190	952	22.10
*Stenotrophomonas* sp. B02	4,199	89.4	65	6	27	0	2,837	2,105	3,367	1,293	3,090	1,036	19.91
*Stenotrophomonas* sp. E09	3,962	89.5	66	7	31	1	2,756	2,059	3,259	1,270	3,021	1,031	17.72
*Streptomyces* sp. G01	8,864	89.7	72	15	11	3	5,381	3,058	7,005	1,703	2,906	1,703	20.93
Un. Bradyrhizobiaceae C05	6,450	87.8	50	3	12	0	4,731	3,250	5,455	1,616	3,158	1,566	16.96
Un. Xanthomonadaceae B08	5,563	88.2	47	1	8	0	2,530	1,794	4,183	1,263	2,491	962	25.51
Un. Xanthomonadaceae G08	4,241	88.8	48	3	5	0	2,993	2,143	3,552	1,299	2,883	1,119	17.33
*Variovorax* sp. F04	6,557	92.8	44	3	12	0	4,850	3,248	5,614	1,602	3,096	1,670	15.37

*Un., Unknown.*

**FIGURE 3:**
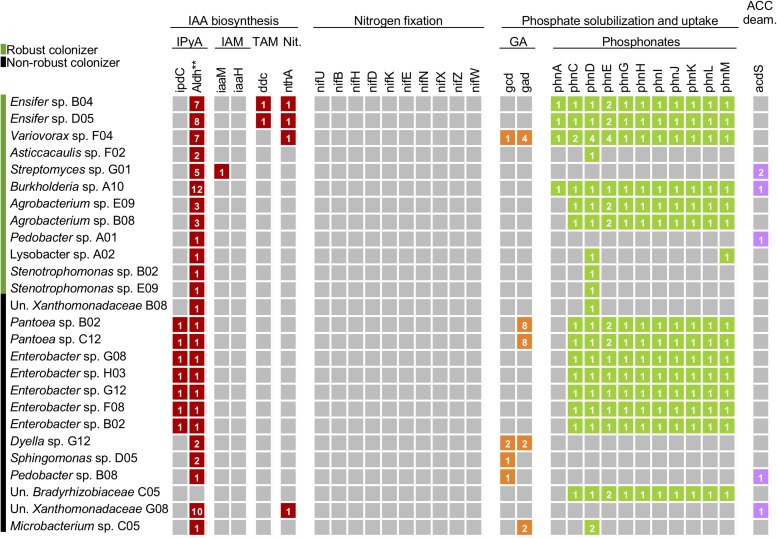
Commonly investigated plant growth-promoting features in plant-associated bacteria are not deterministic of colonization lifestyle. Genomes were surveyed for minimal gene cassettes involved in the biosynthetic pathway of common plant growth-promoting features in bacteria such as indole-3-acetic acid (IAA) production, N fixation, phosphate solubilization and uptake, and ACC deaminase activity. Each column represents a gene of a pathway. The numbers inside colored boxes represent gene copies within a genome. Absence of genes is represented by gray boxes. For IAA production, a total of four different biosynthetic pathways were surveyed (IPyA, IAM, TAM, and nitrilase). Un., Unknown; Nit., Nitrilase; GA, gluconic acid; ACC deam., 1-aminocyclopropane-1-carboxylic acid (ACC) deaminase. Statistical differences in copy number of genes between robust and non-robust colonizer were compared using Student’s *t*-test (^∗∗^*p*-value ≤ 0.01).

We examined whether commonly investigated PGP traits that have been long investigated in isolates from sugarcane are correlated with the colonization efficiency or the magnitude of plant growth promotion observed in the inoculation experiments (Figure 1). This analysis was performed by searching for minimal gene cassettes functionally necessary in the pathways of the IAA production, N fixation, phosphate solubilization and uptake, ACC deaminase activity, and ethylene production (Figure 3). For IAA production, we searched for the genes involved in four different biosynthetic pathways (IPyA, IAM, TAM, and nitrilase pathways). Among the non-robust colonizers, we found that all genomes belonging to the *Pantoea* and *Enterobacter* genera were predicted to have a minimal set of genes for IAA synthesis through the IPyA pathway (Figure 3). Also, the genomes of robust colonizers of the *Ensifer* genus possessed the essential genes for IAA production via nitrilase and TAM pathways, while the genomes of *Stenotrophomonas* sp. E09 and the *Variovorax* and *Agrobacterium* genera possessed genes for the nitrilase pathway. While several putative IAA biosynthetic genes have been reported in the literature, the exact pathway leading to IAA production would need to be further explored in subsequential studies. N fixation is a trait that has long been used for isolation of PGP bacteria from sugarcane. Surprisingly, none of the bacterial genomes of the SynCom included the minimal set of *nif* genes or *nif* clusters necessary for N fixation.

One of the mechanisms by which bacteria promote phosphate uptake by plants involves the production and release of gluconic acid (GA). Among the members of the SynCom, only the robust colonizer *Variovorax* sp. F04 and the non-robust colonizer *Dyella* sp. G12 contain genes encoding the critical enzymes in this pathway, glucose-1-dehydrogenase (Gcd) and gluconic acid dehydrogenase (Gad) (Figure 3). Genes related to phosphonate transport (*phnC*, *phnD*, and *phnE*) and degradation (*phnG*, *phnH*, *phnI*, *phnJ*, *phnK*, and *phnL*) were found in robust colonizers of the genera *Ensifer*, *Variovorax*, *Burkholderia*, and *Agrobacterium*, as well as in the non-robust colonizers of the genera *Pantoea*, *Enterobacter*, and Un. *Bradyrhizobiaceae*. Lastly, genes encoding ACC deaminase were found in three robust colonizers and two non-robust colonizers.

### Genomic Features and Functional Diversity of Robust Colonizers

**FIGURE 4:**
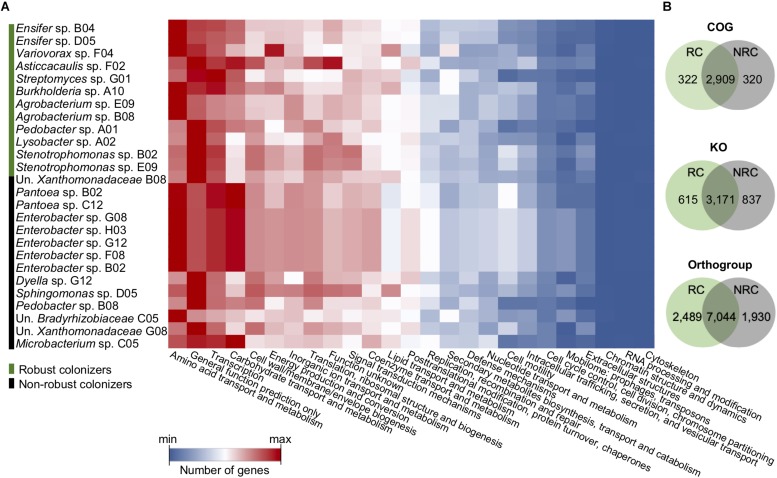
Functional distribution of genes reveals that robust and non-robust colonizer genomes show substantial overlap. **(A)** Heatmap shows gene count per COG functional categories for individual genomes for robust and non-robust colonizers. **(B)** Shared number of COGs, KOs, and orthogroups between robust colonizers (RC) and non-robust colonizers (NRC). Un., Unknown.

To ascertain whether robust colonizers were associated with a specific functional category, we examined the number of genes in each COG category for both robust and non-robust colonizers (Figure 4A). COG annotation was chosen because it has the highest number of functional assignments among proteins. The functional distribution shows that proteins assigned to “amino acid transport and metabolism”, “general function prediction only”, “transcription”, and “carbohydrate transport and metabolism” account, on average, for 35% of all annotated genes in both robust and non-robust colonizers (Figure 4A). No statistically significant association between an entire COG functional category and the colonization lifestyle was found, suggesting that this feature may be correlated to particular functions or an enriched set of functions within the COG categories.

Next, the overlap and exclusivity of functions between robust and non-robust colonizers were evaluated by analyzing COG and KO orthologs between these two groups. The results show a substantial overlap of functions between robust and non-robust colonizers accounting for 82 and 69% of all COG and KO categories, respectively (Figure 4B). However, we identified 322 COGs exclusively found in robust colonizers, 105 of which were associated with the category “function unknown” (Table 3 and Supplementary Table S4). The second most abundant category containing 53 exclusive COGs in robust colonizers was “general function prediction only” (Table 3 and Supplementary Table S4), which is composed mainly of uncharacterized proteins.

**TABLE 3:** Functional overlap and exclusiveness of COGs between robust and non-robust colonizers.

	**COGs**		
	
	**Exclusive to RC**	**Shared**	**Exclusive to NRC**
RNA processing and modification	1	1	0
Energy production and conversion	16	182	27
Cell cycle control, cell division, chromosome partitioning	1	39	9
Amino acid transport and metabolism	8	228	24
Nucleotide transport and metabolism	5	93	7
Carbohydrate transport and metabolism	11	182	28
Coenzyme transport and metabolism	4	167	16
Lipid transport and metabolism	9	95	4
Translation, ribosomal structure, and biogenesis	7	208	7
Transcription	7	108	12
Replication, recombination, and repair	9	111	11
Cell wall/membrane/envelope biogenesis	13	174	10
Posttranslational modification, protein turnover, chaperones	13	141	5
Inorganic ion transport and metabolism	13	191	13
Secondary metabolites biosynthesis, transport, and catabolism	12	66	9
General function prediction only	53	309	33
Function unknown	105	424	77
Signal transduction mechanisms	14	127	12
Intracellular trafficking, secretion, and vesicular transport	7	78	3
Defense mechanisms	6	59	15
Mobilome: prophages and transposons	14	49	15

*Number of shared and exclusive COGs for robust colonizers (RC) and non-robust colonizers (NRC) are shown for each COG functional category.*

Among the protein families that have a functional prediction, we identified a group that is exclusively found in robust colonizers and that are related to plant–microbe interaction such as the beta-glucanases of the GH16 family (COG2273), involved in exopolysaccharide production (Supplementary Table S4). This protein family was found in members of the genera *Agrobacterium*, *Ensifer*, *Asticcacaulis*, *Pedobacter*, *Lysobacter*, and *Streptomyces*. A COG associated with uptake of organic acids by the tripartite ATP-independent periplasmic transporters (TRAP transporters) (COG4664) was exclusively found in the robust colonizers of the genera *Variovorax*, *Agrobacterium*, and *Ensifer*. Interestingly, a COG associated with allantoin metabolism was found in robust colonizers of the genera *Burkholderia*, *Agrobacterium*, and *Ensifer* (Supplementary Table S4). These bacteria displayed the highest relative abundance in the rhizosphere and the endophytic root among the robust colonizers’ members of the SynCom.

To further explore putative functions exclusively associated with the robust colonization lifestyle that might be hidden in unannotated genes, we analyzed groups of clustered proteins based on their homology (orthogroups) with Orthofinder (Emms and Kelly, 2015). Genes without functional prediction had their annotation extended from genes within the same orthogroup. Out of 22,388 identified orthogroups, a total of 9,514 (42.5%) were shared between two or more genomes. Within the shared ones, a total of 8,144 orthogroups had at least one gene with a known Pfam domain or protein prediction based on COG or KO and could be used for extended annotation. We found a total of 2,489 orthogroups that were exclusive to robust colonizers (Figure 4). Several of these proteins were directly related to plant–microbe interaction and might be linked to the robust colonization lifestyle. For example, the OG0002509 protein, which is shared by robust colonizers of the genus *Burkholderia*, *Ensifer*, *Asticcacaulis*, *Agrobacterium*, and *Pedobacter*, was predicted to encode a glycosyltransferase involved in polysaccharide biosynthesis (Supplementary Table S5). Interestingly, proteins involved in the transport of carbohydrates and amino acids were recurrent among orthogroups that are exclusive to robust colonizers. Specifically, we found 56 exclusive orthogroups that were related to the ABC transporter family of sugars and amino acids. These proteins are predicted to transport sorbitol, mannitol, ribose, glucose, and mannose among others. Regarding amino acid metabolism, we found genes encoding transporters related to uptake of branched-chain amino acids, polyamines (spermidine/putrescine), and polar amino acids exclusively in the group of robust colonizers (Supplementary Table S5).

### Enriched and Depleted Functional Families in Robust Colonizers

**FIGURE 5:**
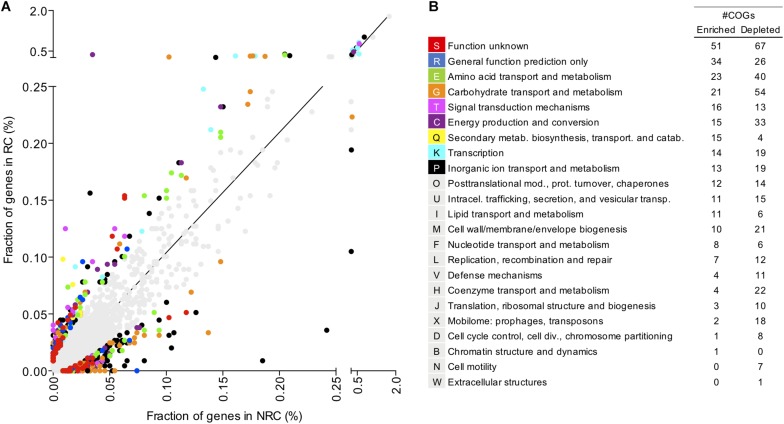
Functional enrichment analysis of COGs shows that specific enriched and depleted functions are associated to robust colonization lifestyle. **(A)** Each dot in the graph represents a distinct COG and the fraction of genes associated with that COG in robust colonizers (RC) and non-robust colonizers (NRC). Fraction of genes was determined as the number of genes assigned to a given COG divided by the total number of genes in RC and NRC. Colored dots represent COGs that are significantly (*P* < 0.05) enriched or depleted in robust colonizers. Gray dots are COGs not significantly enriched or depleted. **(B)** Number of COGs significantly enriched or depleted in the robust colonizers. COG names were shortened when necessary. Enriched and depleted COGs are listed in Supplementary Table S6.

**FIGURE 6:**
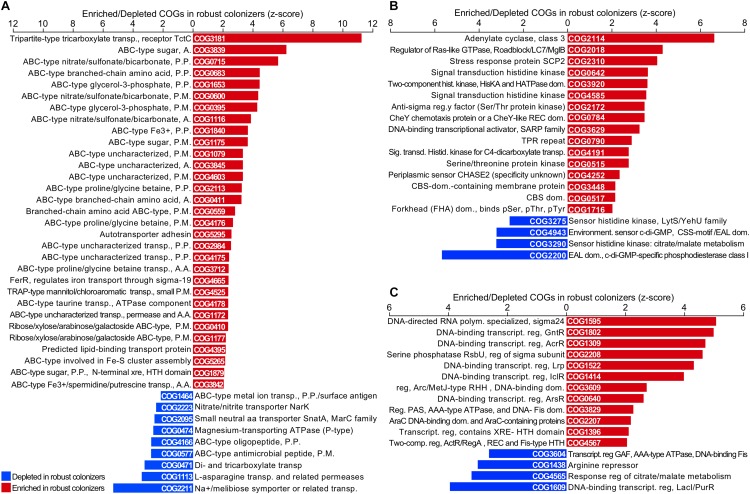
Enrichment and depletion of COGs associated to transporters **(A)** signal transduction mechanisms **(B)** and transcription **(C)** in robust colonizers. Only COGs significantly enriched or depleted are displayed (*P* < 0.05). Original COG IDs were maintained and names were shortened when necessary.

Given that a substantial fraction of protein families is shared between robust and non-robust colonizers, we investigated whether colonization success is connected to differential enrichment or depletion of specific functions. This question was addressed by performing an enrichment analysis of gene count per COG in the robust and non-robust colonizers. A total of 276 COGs was found to be significantly enriched in robust colonizers (Figure 5). Surprisingly, genes from enriched COGs accounted for 22% of the total gene fraction in robust colonizer’s genomes. Although most of these COGs belong to the category of “function unknown” and “general function prediction only”, gene frequency was highly enriched in COGs associated with the categories of “carbohydrate transport and metabolism”, “amino acid transport and metabolism”, “signal transduction mechanisms”, and “transcription”. Genes in these categories accounted for 3.9% of total annotated proteins in robust colonizers and <1% of total annotated proteins in non-robust colonizers (Figure 5). To further investigate enriched functions in robust colonizers, we compared the rank of enriched COGs by gene count and the rank of enrichment level as determined by *z*-score. A total of 40 COGs that were found in both ranks were associated mainly with carbohydrate and amino acid transporters (Figure 6A). In total, genes annotated as transporters accounted for 3.4% of total annotated proteins in robust colonizers. Specifically, COGs assigned to periplasmic ATPase and permease components of ABC-type transporters were highly enriched in all robust colonizer genomes (Figure 6A and Supplementary Table S6).

Regarding carbohydrate metabolism, inspection of KEGG functional annotation revealed that enriched transporters were related to the uptake of a broad range of organic acids and sugars such as succinate, malate, fumarate, sorbitol, mannitol, maltose, arabinose, and monosaccharides (Figure 6A and Supplementary Table S6). The tripartite-type tricarboxylate transporter system was found exclusively in *Lysobacter*, *Agrobacterium*, *Ensifer*, and *Variovorax* (Supplementary Table S6).

Out of 23 enriched COGs from the “amino acid transporters and metabolism” category, a total of 9 COGs were involved in amino acid transport. ABC transporter for branched-chain amino acids accounted for 1.3% of total annotated proteins in robust colonizers (Figure 6A and Supplementary Table S6). Proteins related to ABC-type transporters of glycine betaine/proline, branched-chain amino acids, spermidine/putrescine, neutral amino acids, and histidine, as well as threonine/homoserine/homoserine lactone efflux proteins were enriched in robust colonizers. Curiously, genes of the same category that are involved in the biosynthetic pathway of several amino acids including proline, histidine, serine, asparagine, methionine, arginine, lysine, and tryptophan were depleted in robust colonizers compared to the non-robust colonizers.

A total of 16 enriched COGs that belong to signal transduction mechanisms and 14 that belong to transcription were enriched in robust colonizers, mostly related to kinase receptor systems (Figures 6B,C and Supplementary Table S6). Close inspection of KEGG and Pfam domains of these kinases revealed that they are related to perceiving carbon (C) availability, nutrient uptake, motility, and response regulators to environmental stresses such as osmotic stress. Among the transcription regulators, we found enriched families related to biofilm formation, biosynthesis of antibiotics, response to osmotic stress and toxic chemicals, and pathogenicity.

## Discussion

### Genome Sequencing of Bacterial Genomes From a Plant Growth-Promoting SynCom

Plant microbiome profiling has revealed rich and diverse communities of bacteria and fungi that may be important for the growth and health of their hosts (Karthikeyan et al., 2007; Lundberg et al., 2012; Bulgarelli et al., 2015; Zarraonaindia et al., 2015; Castrillo et al., 2017). Although sequencing of ribosomal genes and metagenomes has enabled the discovery of communities’ assemblage dynamics, a deep understanding of mechanisms involved in plant–microbe interaction and microbiota functions that benefit plant growth still requires methods to systematically isolate and cultivate microorganisms for their use in controlled experiments, or to assess their encoded function by sequencing (Lebeis et al., 2012; Bai et al., 2015; Browne et al., 2016; Liu et al., 2017).

An emerging approach that supports large-scale isolation of the microbiota is the community-based culture collection, which consists of picking colonies regardless of whether they contain single or multiple microorganisms (Armanhi et al., 2016). Because isolates from a community-based culture might contain multiple microorganisms, sequencing and assembly of isolated communities might result in metagenomes. In such cases, retrieval of individual high-quality genomes from metagenome sequences requires approaches to bin individual genome sequences confidently.

Binning high-quality genomes from metagenomes has been a significant challenge in microbiome studies (Albertsen et al., 2013; Lin and Liao, 2016). Here, to bin individual genomes from community isolates, we applied a combined strategy based on grouping sequences based on genomic signature, markers genes, sequencing coverage estimates, and taxonomic affiliation. These approaches have been used independently and jointly to successfully bin genomes from metagenomes of different environments and with different level of diversity (Lin and Liao, 2016). Specifically, binning by coverage significantly enhanced discrimination of individual genomes in our data. In addition, the use of PCR-free methods to prepare sequencing libraries contributed to the evenness of coverage in sequences belonging to the same genome and significantly improved the binning process. The binning approach used in this work allowed assembly of 26 individual genomes from the 17 community-based isolates comprising a SynCom that displayed a completeness estimate of above 98% and contamination below 2%.

Our data suggest that reconstruction of individual genomes from community-based isolates is possible by employing approaches of differential binning based on both sequence composition and coverage. Moreover, these results validate the use of the community-based isolates in functional and structural genomic analysis.

Genomes and metagenomes IDs are listed in Supplementary Table S7. The full genome sequence of community-based isolates (metagenomes), isolated genomes, and gene annotation of each genome and metagenome are available in the IMG database, https://img.jgi.doe.gov/. Genomes and metagenomes IDs were simplified in the manuscript text for clarity purposes. The original ID, as deposited in the IMG database, can be found in Supplementary Table S7. Raw data of community profiling by 16S rRNA gene sequencing of inoculated plants were deposited at Sequence Read Archive (SRA) database under the accession number SRP126483.

### Genome Annotation of the SynCom Reveals That Robust Colonization Does Not Correlate With Common PGP Traits

Isolation of microbial communities from plants has traditionally been done by the isolation of microbes that display well-known PGP activities, such as phytohormone production or N fixation. Despite the massive number of studies about these commonly investigated features, there is limited evidence about the magnitude of their contribution to plant growth under natural conditions or their relatedness with the establishment of a successful association between plants and microorganisms (Barret et al., 2011; Bulgarelli et al., 2013; Finkel et al., 2017). In fact, recent studies of microbial diversity associated with plants show that highly abundant groups have been neglected (Vandenkoornhuyse et al., 2015; de Souza et al., 2016; Sánchez-Cañizares et al., 2017). In this work, by sequencing the bacterial genomes from a SynCom which was assembled based on the abundance of microbial groups *in planta* and proven to benefit plant growth (Armanhi et al., 2018), we have addressed whether commonly studied PGP features correlate with bacterial colonization efficiency.

Several bacterial species can stimulate plant growth either by inducing plants to produce IAA or by producing bacterial IAA itself (Bastián et al., 1998; Thakuria et al., 2004; Wani and Khan, 2010; Bal et al., 2013). Because the exogenous supply of IAA increases plant susceptibility to bacterial infection, it has been suggested that IAA is a primary signaling molecule for successful plant–microbe association (Spaepen and Vanderleyden, 2011; Sukumar et al., 2013). However, there is no evidence that bacterial IAA production contributes to improved bacteria plant association or whether it is determinant for successful bacterial colonization. Here, we verified that most of the robust colonizers of our SynCom do not have the minimal gene set required for IAA biosynthesis. On the other hand, among the non-robust colonizers, representatives of the genus *Enterobacter* do contain a minimal gene set for IPyA, the most common pathway of IAA production in bacteria. These results suggest that IAA production is not a necessary feature for robust colonization.

Molecular N fixation has been well documented in sugarcane. The interest in N fixation comes from evidences suggesting that sugarcane varieties are capable of obtaining large amounts of N from biological fixation (Urquiaga et al., 2012). Numerous studies have isolated N-fixing bacteria from the rhizosphere of these plants using selective media (Döbereiner et al., 1972; Sevilla et al., 2001; Baldani et al., 2002). These studies have primarily targeted diazotrophic groups of the genera *Beijerinckia*, *Gluconacetobacter*, *Azospirillum*, and *Herbaspirillum* (Cavalcante and Döbereiner, 1988; Baldani et al., 1992; James et al., 1994; Olivares et al., 1996; Reis, 2004). Surprisingly, none of these bacteria have been found in significant abundance in sugarcane (de Souza et al., 2016). In addition, none of the bacterial genomes of our SynCom contain genes related to N fixation. This finding strongly correlates with studies of the *Rhizobiales* groups showing that the majority of isolates from roots lack N-fixing capability (Garrido-Oter et al., 2018).

One of the mechanisms by which bacteria improve plant acquisition of phosphate is by producing and releasing gluconic acid (GA). GA acts by solubilizing poorly soluble minerals making them available to plants. The Gcd and Gad are critical enzymes in the biosynthetic pathway of GA and have been widely documented in many P-solubilizing bacteria (Stella and Halimi, 2015). Among the robust colonizers of our SynCom, the *gcd* and *gad* genes were found only in *Variovorax* sp. F04, demonstrating a weak association of this feature with the robust colonization lifestyle. A second mechanism commonly documented in solubilizing bacteria is the degradation of phosphonates that are compounds containing chemically stable C–P (Singh and Satyanarayana, 2011). Some bacteria contain genes encoding enzymes capable of breaking these bonds and releasing P in a form readily absorbed by plants (Chuang et al., 2007; Poonguzhali et al., 2008; Ahemad and Khan, 2010). Notably, both robust and non-robust colonizers from our SynCom contain gene cassettes for phosphonate metabolism and transport, suggesting that this feature is not determinant for colonization efficiency.

Overall, our results show that commonly studied PGP traits are not determinant factors for robust colonization. In fact, given that most of the robust colonizers do not contain features for IAA production, N fixation, or ACC deaminase, it is likely that the growth promotion observed in inoculation experiments is due to other features encoded by their genomes. Furthermore, given that most approaches for searching plant growth-promoting microorganisms are based on pre-screening methods of these traits, our data certainly raise the question as to whether other important microbial features are being overlooked. Additionally, our data show that common PGP traits might not be suitable to predict plant phenotypes originated from interaction with microbes or features related to microorganism colonization and persistence in plants. More importantly, it suggests that mechanisms in plant–microbe interaction and its outcomes await discovery.

### Functions Associated With Robust Colonization Lifestyle

Comparative genomic analyses of bacterial members of our SynCom revealed substantial overlap of functions between robust and non-robust colonizers. It is likely that the functional overlap may reflect the ability of bacteria in the SynCom to colonize and live in the same niche. This assumption is consistent with a functional overlap found in root and leaf microbiota (Bai et al., 2015), and might indicate bacterial adaptation by convergence to the plant environment.

Among the exclusive protein families encoded by the genomes of robust colonizers, we found genes related to the production of exopolysaccharides, specifically beta-glucanases, found in members of the genera *Agrobacterium*, *Ensifer*, *Asticcacaulis*, *Pedobacter*, *Lysobacter*, and *Streptomyces*. Exopolysaccharides have been linked to infection and colonization processes by supporting bacterial attachment to the root surface (Marczak et al., 2017). In rhizobia, for example, secreted exopolysaccharides build a biofilm that increases nutrient absorption and enhances communication between plant and bacteria (Ghosh and Maiti, 2016; Marczak et al., 2017). Also, mutants of *Gluconacetobacter diazotrophicus* lacking the enzymes for polysaccharide production are defective in endophytic colonization of sugarcane roots (Meneses et al., 2011). The fact that genes for biosynthesis of exopolysaccharides were found exclusively in most robust colonizers suggests that this trait may play a pivotal role in sustaining the robust colonization lifestyle.

Some protein families related to transport of sugars and amino acids were also exclusively found in robust colonizers. In the case of sugars, the tripartite ATP-independent periplasmic transporters (TRAP transporters) were found in robust colonizers of the genera *Variovorax*, *Agrobacterium*, and *Ensifer*. These transporters are responsible for the uptake of organic acids such as succinate, malate, and fumarate, all of which are found in root exudates of maize and sugarcane (Singh and Mukerji, 2006). It is likely that these transporters help robust colonizers to efficiently acquire C compounds provided by the plant, which would benefit competition for resources and ultimately increase microbe fitness for a robust colonization lifestyle.

Sugar transport was a prominent type of function highly correlated to the robust colonization lifestyle. The comparative genomics analysis revealed that robust colonizers had 7.4-fold more genes related to sugar transport than the non-robust colonizers. Because these transporters are linked to the uptake of a broad range of sugars, from monosaccharides to organic acids, it is likely that the diversity of transporters might be related to the nutritional complexity of soil environment and root exudates. Given that plant exudates vary among plant species and plant nutritional status (Bais et al., 2006; Singh and Mukerji, 2006; Turner et al., 2013), by maintaining a broad repertoire of transporters the bacteria might efficiently capture resources from different types of hosts and plant organs and successfully compete during plant colonization.

Amino acid transporters were also enriched in the robust colonizers. Specifically, transporters linked to the uptake of branched-chain amino acids were found among exclusive and enriched proteins of robust colonizers. The supply of branched-chain amino acids has been reported to be essential for growth, N fixation, and persistence of rhizobia (Prell et al., 2009). Because robust colonizers devote a large extent of genetic resources in the uptake of branched-chain amino acids, they might also play an import role as regulators of non-nodulating plant–microbe association. Therefore, the efficient capture of amino acids from plant exudates is likely to provide economy of resources by inactivating their biosynthetic pathways, which ultimately enhances microbe fitness.

Finally, we identified a set of functions that are highly correlated to the successful establishment of bacterial communities in plants. While some of the enriched function in robust colonizers belong to known metabolic pathways, such carbohydrate and amino acid metabolism, many are unknown and represent new roles that should be the subject of further work.

## Data Availability

Genomes and metagenomes IDs are listed in Supplementary Table S7. The full genome sequence of community-based isolates (metagenomes), isolated genomes, and gene annotation of each genome and metagenome are available in the IMG database, https://img.jgi.doe.gov/. Genomes and metagenomes IDs were simplified in the manuscript text for clarity purposes. The original ID, as deposited in the IMG database, can be found in Supplementary Table S7. Raw data of community profiling by 16S rRNA gene sequencing of inoculated plants were deposited at Sequence Read Archive (SRA) database under the accession number SRP126483.

## Author Contributions

PA, JI, and RS coordinated the project. JA and RS designed the SynCom and performed the inoculation experiments with the support of ND. JA prepared sequencing libraries of 16S rRNA gene for microbial profiling. RS prepared the genome sequencing libraries and performed the genome assembling and bioinformatic analysis. All authors read and approved the final manuscript.

## Conflict of Interest Statement

The authors declare that the research was conducted in the absence of any commercial or financial relationships that could be construed as a potential conflict of interest.

## Footnotes

1 https://img.jgi.doe.gov/
